# Intraspecific Trait Variation in Tree Species Responds to Environmental Heterogeneity at Range‐Wide but Not Local Scales

**DOI:** 10.1002/ece3.72224

**Published:** 2025-09-28

**Authors:** Eva Arroyo, Robert Muscarella, María Uriarte

**Affiliations:** ^1^ Department of Ecology, Evolution & Environmental Biology Columbia University New York New York USA; ^2^ Plant Ecology and Evolution, Department of Ecology and Genetics Uppsala University Uppsala Sweden

**Keywords:** Bayesian statistics, climate gradients, community ecology, functional ecology, functional traits, intraspecific trait variation, ITV, trait‐environment interactions, tropical forest ecology

## Abstract

Theoretical and empirical ecology have recently explored the role of intraspecific trait variation (ITV) in structuring ecological communities. Such trait variation may be correlated with high environmental variability within a site or across a species' range. Here we explore the relationship between ITV, spatial and temporal environmental heterogeneity, and the breadth of species' environmental distributions for 33 tree species across eight sites occurring along marked environmental gradients in Puerto Rico. Specifically, we asked (1) If within‐site ITV is positively correlated with site‐level temporal and spatial environmental variation, and (2) How across‐site ITV relates to the breadth of species' environmental distributions on the island. Two key plant traits, leaf mass per area (LMA) and wood density, were used to understand the association between ITV and environmental variation. We examined ITV across species and sites using statistical models to assess the relationship between (1) within‐site ITV and site‐level spatial (topography) and temporal (rainfall) environmental variability; (2) across‐site ITV and environmental variation across the species geographic range on the island. We also assessed the relationship between across‐site mean trait values and island‐wide environmental ranges. (1) Across all species, we did not find any significant associations between within‐site ITV and site‐level temporal or spatial variability. (2) Greater ITV values of LMA and wood density were associated with larger environmental ranges. We also found that species with high wood density, a trait associated with a conservative growth strategy, had narrower ranges across climatic conditions, but this pattern was not evident for LMA. Our findings emphasize the complexity of the relationships between ITV and species distributions with respect to environmental heterogeneity at different spatial and temporal scales. These complexities are important for research on species distributions and range‐shift dynamics.

## Graphical abstract

Here we explore the relationship between ITV, spatial and temporal environmental heterogeneity, and the breadth of species' environmental distributions for 33 tree species across eight sites across marked environmental gradients in Puerto Rico. Species with greater across‐site ITV in wood density and LMA occurred over broader ranges of potential evapotranspiration but lower elevation ranges. We also found that species with high wood density, a trait associated with a conservative growth strategy, had narrower ranges across climatic conditions, while there were no consistent patterns for species with high LMA.

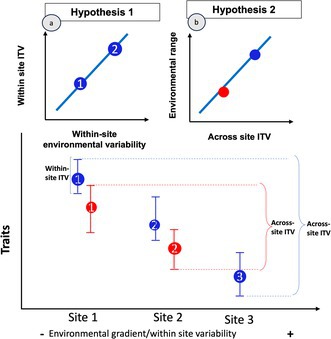

## 1 Introduction

Despite clear evidence showing that species ranges are determined by habitat suitability (Lee‐Yaw et al. 2016; Wisz et al. 2013), the reasons why some species have broad environmental ranges and some narrow ones remain elusive (MacArthur 1957; Rabinowitz 1982; May and Frs 1999; Gaston 2009; Boyd et al. 2022). Is it that some species are more variable such that they can express phenotypes suitable for a wider range of environmental conditions, or is it that some ecological strategies confer an ability to occupy a wider range of environmental conditions? In addition, because fine‐scale data on environmental variability is often not incorporated in species occurrence modeling, it is difficult to know if species' presence at a site results from specialization on suitable patches within environmentally variable sites (Bruelheide et al. 2018; but see De Frenne et al. 2021; Haesen et al. 2023), or if phenotypic variation allows species to better cope with highly variable sites (Angert 2009; Buckley et al. 2010; Eckert et al. 2008; Freestone et al. 2011; Guisan and Rahbek 2011; Valladares et al. 2014).

In highly diverse systems with long‐lived organisms (e.g., tropical forests), functional traits help infer species' responses to their environment (McGill et al. 2006; Díaz et al. 2016; Laughlin 2023). Two key tree traits are leaf mass per area (LMA; g cm^−2^) and wood density, which reflect light and water use strategies, respectively. LMA, central to the leaf economics spectrum, tends to be positively correlated to nutrient content, photosynthetic rate, and leaf life span (Westoby et al. 2004). High LMA indicates slower photosynthetic rates and lower mortality, and conservative resource use (Westoby et al. 2004; Reich 2014), often under conditions of limited light and water availability. Low LMA, in contrast, is associated with acquisitive strategies, common in wetter, more fertile soils, and higher light environments (Markesteijn and Poorter 2009; Poorter et al. 2009). Wood density (g cm^−3^) also tends to be negatively correlated with many of the “fast” growth traits: species with high wood density tend to have lower rates of maximum photosynthesis, growth, and water uptake, but more resistance to xylem embolisms and physical damage (Swenson and Enquist 2007; Reich 2014), aiding drought and pest resistance and facilitating growth in the shade (Carlquist 1977; Hacke and Sperry 2001; Reich 2014).

Traits are often discussed and analyzed using species mean values, but variation within species, that is, intraspecific trait variation (ITV), reflects an important component of phenotypic variation, the magnitude of which often matches or exceeds that of interspecific variation (Messier et al. 2010; Violle et al. 2012; Siefert et al. 2015). Incorporating ITV into analyses improves our understanding of species association with their current environment, as well as their potential responses to future environmental change (Siefert 2012; Siefert et al. 2015; Violle et al. 2012, though see Cardou et al. 2022 and Fajardo et al. 2024). ITV may have additional impacts on community structure, with some authors suggesting added plasticity may strengthen coexistence, and others that it may destabilize it (Stump et al. 2021; Bolnick et al. 2011; Turcotte and Levine 2016). Nonetheless, relatively few studies to date have directly assessed the magnitude of ITV in diverse communities, the distribution of ITV at different spatial scales, and the relationship of ITV with spatial and environmental heterogeneity.

**FIGURE 1:**
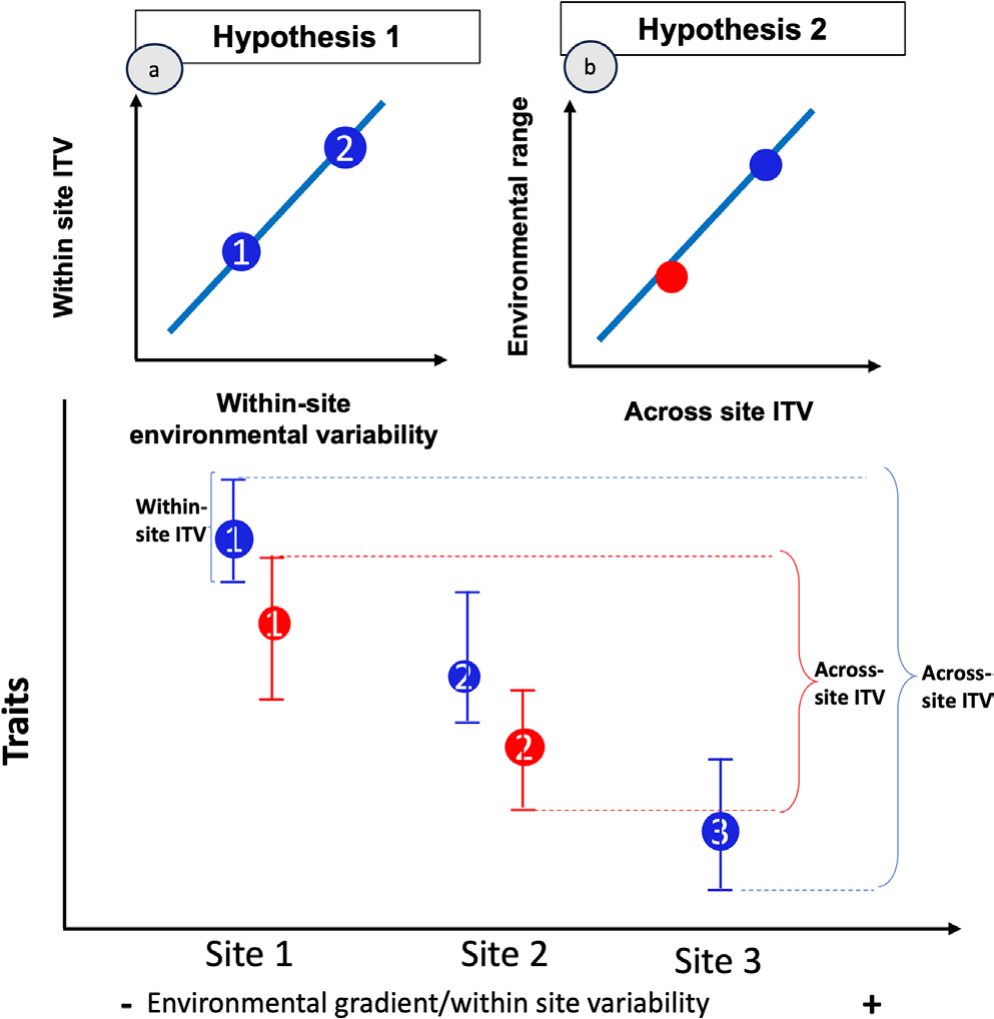
Expected directions of relationships for Hypotheses 1 (a) and 2 (b). The bottom figure shows the distinction between within‐ and across‐site ITV.

Within any one site, ITV may be larger at sites with more spatial or temporal environmental variability (Figure 1). Temporal variation within a site, such as rainfall seasonality, can increase the range of conditions species experience and may thus promote within‐site ITV. For example, studies have found variation in hydraulic traits over time in response to changing rainfall conditions (Anderegg 2015; Trugman et al. 2020). ITV may also be higher at sites with more spatial variability in environmental conditions. For example, terrain roughness can lead to spatial variation in soil water and nutrient availability. Indeed, some studies have found higher interspecific trait variability in grassland species in sites with high spatial heterogeneity in soil depth (Price et al. (2017)) and more variable soil chemistry (Baythavong (2011)), and we expect similar patterns for intraspecific variation.

Across sites, greater ITV may reflect a wider niche breadth, either because phenotypically variable species might tolerate a wider range of environments or because broader environmental ranges drive that variation due to plasticity (Figure 1). In a meta‐analysis of 66 alpine species, species with higher ITV occurred across broader environmental ranges (Rixen et al. 2022). ITV may thus allow local trait–environmental matching (Valladares et al. 2014; Fajardo et al. 2024). However, higher phenotypic variation may not always signal broader niche breadth (Sultan 2001). Sometimes, especially those reflecting conservative strategies such as denser wood or higher LMA, better predict niche breadth. Such traits may confer resilience in harsh conditions, enabling broader environmental occupancy even with limited ITV (Reich 2014) (Valladares et al. 2007). With conservative mean trait values (e.g., high wood density or LMA), limited phenotypic variation may actually enhance overall fitness across varying environments—allowing low‐ITV species to occupy broader ecological ranges more successfully.

**FIGURE 2:**
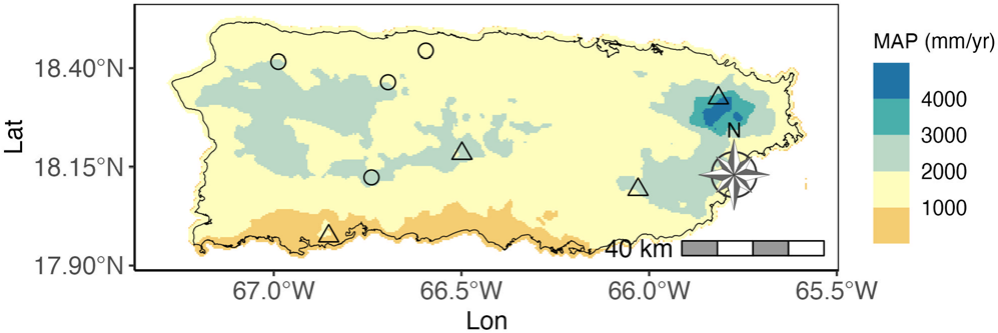
Locations of the eight study sites on volcanic (circles) or limestone (triangles) soil and Mean Annual Precipitation (MAP).

Here we use occurrence and trait data for 33 tree species collected at 8 sites across environmental (climate and geology) gradients on the island of Puerto Rico (Figure 2) to examine the associations between within‐ and across‐site ITV and environmental heterogeneity at site and island scales. We focus on wood density and LMA because of their physiological relevance to water and light resource strategies, exhibit high ITV (Fajardo et al. 2024), and have been extensively studied in forests (Westoby et al. 2004; Poorter et al. 2009; Wright et al. 2010; Reich 2014), including in Puerto Rico (Muscarella et al. 2016). We use this data to test the following hypotheses:

1. At sites with higher environmental heterogeneity, species will have higher within‐site ITV than at sites with low environmental variation. Specifically, we expect higher within‐site ITV at sites with more topographic heterogeneity (spatial variability) and at sites with more seasonal rainfall (temporal variability) (Figure 1a).
2. Species with broader environmental ranges will have more variable traits (Figure 1b). We expect higher across‐site ITV in wood density to be associated with a broader range of water variability, as reflected by climatic water indices and geology. Because low LMA is associated with acquisitive light use strategies, we expect species with high LMA ITV to occur across a greater range of solar irradiance.

An alternative hypothesis is that conservative traits (high species mean values of wood density and LMA) could lead to greater ability to withstand a broader range of environmental conditions. If this is the case, we expect species with conservative trait values to have lower across‐site ITV and occur across a wider range of environmental conditions.

## 2 Methods

### 2.1 Study Site

Puerto Rico is a Caribbean island in the Greater Antilles (9104 km^2^) with marked precipitation, elevation, and geological gradients, resulting in six Holdridge life zones from subtropical dry forest to subtropical rainforest (Ewel and Whitmore 1973). Volcanic and limestone geologies dominate the island, although alluvial and ultramafic soils are also present (Miller and Lugo 2009). Volcanic soils tend to hold more water and be more nutrient‐rich, whereas limestone soils tend to be better drained and poorer in nutrients (Camacho 2005; Miller and Lugo 2009). Elevation ranges from 0 to 1338 m above sea level, and mean annual rainfall ranges from 800 to 4590 mm (Figure 2). Limestone soils span a narrower precipitation gradient (752–2229 mm/year) than volcanic soils (1188–4590 mm/year). Our eight study sites capture much of this environmental variation (Table 1, Figure 2).

**TABLE 1:** Site information and number of species with trait data at each site.

Site	MAP	Geology	Elevation (m)	Rainfall variability	Topographic variability	# Species wood density	# Species for LMA
Cambalache (CAM)	2351.10	V	82.00	28.31	5.40	12	10
Carite (CAR)	1545.40	L	577.90	38.25	2.85	15	15
Guajataca (GUA)	2068.90	L	221.50	41.71	6.24	14	12
Guanica (GUN)	2139.80	V	878.30	54.12	5.61	20	19
Guilarte (GUI)	1861.90	V	186.00	59.41	3.13	5	3
Luquillo (LUQ)	1728.50	L	1090.80	26.85	3.59	28	27
Rio Abajo (RIO)	2220.10	V	317.00	41.61	7.12	15	15
Toro Negro (TOR)	1039.30	L	887.30	44.07	3.62	19	19

### 2.2 Species Data

Environmental range data for each tree species were derived from 17,479 occurrence records compiled from herbaria and forest plot data sets (Muscarella and Uriarte 2016). The study sites in which trait data were collected do not cover the full geographic range of the study species, so we use this occurrence data from herbaria to derive environmental range data rather than the plot data used for trait modeling (see below).

Trait variation and species abundance data come from eight, 0.25 ha forest plots established in 2014 at eight forest reserves (Table 1, Figure 2). Forests at all sites were older than 80 years at the time of sampling. We restricted our analyses to 33 species that occur at > 3 of the 8 sites, with > 5 individuals per site (Table A1). These thresholds were chosen based on available data in order to ensure that trait estimates were based on a minimally sufficient sample size while retaining enough species for analysis. For each of these 33 species, we collected functional trait data for wood density (g/cm^3^) and LMA (g/cm^2^) by sampling individuals within each field plot (Muscarella and Uriarte 2016; Muscarella et al. 2016). Data were collected between 2017 and 2019. For LMA, we collected sun‐exposed and fully mature leaves. LMA varies substantially from sun‐exposed to shaded leaves (Legner et al. 2014; Paź‐Dyderska et al. 2020), so by sampling only sun‐exposed leaves, we limited measured ITV for LMA. Wood density was measured on branches of approximately similar diameters (1–2 cm). Multiple traits may be needed to understand the environmental and competition responses of species (Clark et al. 2010; Muscarella and Uriarte 2016; Uriarte et al. 2018), but because ITV of wood density and LMA are uncorrelated in our dataset (Pearson's correlations, *p* = 0.06, *r* = −0.33), we analyze them separately.

### 2.3 Statistical Analyses

Sample sizes varied substantially across sites and species, so we used Bessel's correction to correct for the effect of sample size on variance (Upton and Cook 2014). The sample‐size corrected variance for a given trait, (ITV*) of species *sp* within a site *si*, is given by:

(1a) $${\mathrm{ITV}}_{\mathrm{si},\mathrm{sp}}^{*}=\frac{n}{n-1}\;{\mathrm{ITV}}_{\mathrm{si},\mathrm{sp}}$$

and the ITV* of a species across all sites is:

(1b) $${\mathrm{ITV}}_{\mathrm{sp}}^{*}=\frac{n}{n-1}\;{\mathrm{ITV}}_{\mathrm{sp}}$$

These data are distributed log‐normally, so we log transformed the trait values prior to analyses. This transformation still results in logarithmically distributed ITV* values, so in all cases, models use the log ITV* as response variables. Additionally, by using this transformation, ITV* is identical for a trait and its inverse. This is especially relevant for LMA, where its inverse (specific leaf area) is often used as an analogous ecological indicator.

To address Hypothesis 1, that within‐site ITV is higher in sites with more topographic or rainfall variability, we fitted mixed‐effects models with random slopes for the relationship between ITV*_si,sp_ and environmental variability. We fitted separate models for topographic and rainfall variability. We obtained topographic data from the 5 m USGS 3DEP DEM (2017) and calculated topographic variability within a site (${\text{roughness}}_{\mathrm{si},\mathrm{sp}}$) using the site‐level mean for the terrain roughness index at the 5‐m scale. We used the *terra* package in ArcGIS for these calculations (Reily et al. 1999). If the terrain roughness is very high for a cell, this means it is a patch that is very high elevation surrounded by lower elevation (or low elevation surrounded by higher elevation). A plot with many high‐terrain roughness values is uneven. Geology data was obtained from Bawiec (1998).

Long‐term averages for climate data were obtained from a variety of sources. Intra‐annual rainfall variability was calculated as the coefficient of variation in precipitation across months, ${\text{cvppt}}_{\mathrm{si},\mathrm{sp}}$ at each site, using rainfall data from CHELSA Version 2.1 (Karger et al. 2017). This dataset provides high‐resolution (~1 km) average precipitation data derived from the period 1979–2013. Mean PET and CWD were also obtained from CHELSA Version 2.1 (Karger et al. 2017) Cloud cover was obtained from EarthEnv (Wilson and Jetz 2016), which uses 15 years of twice‐daily MODIS satellite images integrated over the time range of 2000–2014. Solar irradiance from the *Global Solar Atlas 2.0* (ESMAP 2019), which uses long‐term averages for solar resource between 1994 and 2024 at ~250 m resolution.

For each of the two models, ITV in site *si* for species *sp* takes the form:

(2a) $$\log\left({{\mathrm{ITV}}^{*}}_{\mathrm{si},\mathrm{sp}}\right)=\beta_{0}+\beta_{1,\mathrm{sp}}+\beta_{2,\mathrm{sp}}\times \text{environmental variabilit}y_{\mathrm{si}}+\epsilon /n_{\mathrm{si},\mathrm{sp}}$$

(2b) $$\beta_{1,\mathrm{sp}}\sim \text{Normal}\;\left(\mu_{1},\sigma_{1}\right)$$

(2c) $$\beta_{2,\mathrm{sp}}\sim \text{Normal}\;\left(\mu_{2},\sigma_{2}\right)$$

(2d) $$\epsilon \sim \text{Norm}\left(0,\tau \right)$$

This model includes fixed and random effects for species (Table 2): $\mu_{1}$ is the estimated ITV for a trait, across all species; $\mu_{2}$ is the estimated average effect of environment on ITV across all species; $\beta_{1,\mathrm{sp}}$ estimates species average phenotypic variation (a species random effect for ITV) across the sites; $\beta_{2,\mathrm{sp}}$ estimates the species‐specific effect of environmental variability on ITV (a random slope on the relationship between within‐site ITV and environmental variability). Standard deviations ($\sigma_{1}$, $\sigma_{2}$) were given uniform priors (0, 10) and means and intercept ($\beta_{0},\mu_{1},\mu_{2}$) normal (0,1) priors. The error of the predictions was scaled to sample size ($n_{\mathrm{si},\mathrm{sp}}$), such that larger sample sizes result in smaller predicted error.

**TABLE 2:** Variable descriptions for Equations ((2a), (2b), (2c), (2d)).

Variable	Description
ITV_si,sp_*	The sample‐size adjusted trait variation at a particular site for a particular species.
$\beta_{0}$	The intercept of the model across all species and sites
$\beta_{1,\mathrm{sp}}$	The species‐specific intercept (random intercepts)
$\beta_{2,\mathrm{sp}}$	The species‐specific slope between environmental variability and ITV* (random slopes)
$\mu_{1}$	Estimated average intercept across all species
$\mu_{2}$	Estimated average slope between environmental variability and ITV* across all species
ϵ	Error term

Hypothesis 1 predicts a positive association between environmental variability and ITV* ($\mu_{2}$) across all species. For this hypothesis, we also examine species‐specific responses to rainfall and terrain variability ($\beta_{2,\mathrm{sp}}$).

To address Hypothesis 2, that species with broader environmental ranges will exhibit higher levels of across‐site ITV, we calculated the range (95% confidence interval across occurrence records) for elevation, potential evapotranspiration (PET; mm/year), climatic water deficit (CWD; mm), and irradiance across the occurrence dataset mentioned above (Muscarella and Uriarte 2016). We also considered the number of geological classes in which a species occurs to represent its geological range. PET and CWD were obtained from CHELSA Version 2.1 (Karger et al. 2017), cloud cover was obtained from EarthEnv (Wilson and Jetz 2016), geology data from Bawiec (1998), and solar irradiance from the *Global Solar Atlas 2.0* (ESMAP 2019).

For this analysis, we use the across‐site ITV (all trait data within a species). Our model takes the form:

(3) $$\begin{array}{cc} \log\left({{\mathrm{ITV}}^{*}}_{\mathrm{sp}}\right)= & \beta_{1}+\beta_{2}\;\mathrm{PET}\;{\text{range}}_{\mathrm{sp}}+\beta_{3}\mathrm{CWD}\;{\text{range}}_{\mathrm{sp}}\\ & +\beta_{4}{\text{geological range}}_{\mathrm{sp}}+\beta_{5}\text{elevation}\;{\text{range}}_{\mathrm{sp}}\\ & +\beta_{6}\text{irradiance}\;{\text{range}}_{\mathrm{sp}}+\beta_{7}\text{cloudiness}\;{\text{range}}_{\mathrm{sp}} \end{array}$$

Many of these predictors were correlated; therefore, we carried out model selection using AIC and report VIF for the final models as well as the correlation matrix (Supplement 1, Figure S1). Because these predictor variables are on different scales, all were normalized with mean 0, standard deviation 1 prior to analyses. Equation (3) is a non‐hierarchical Bayesian model, run in R using the *arm* package (Gelman and Su 2021). We evaluated the effect of each predictor using 90% credible intervals.

If more conservative traits, rather than ITV, lead to larger range size (our alternative hypothesis), we have two expectations. First, we expect species with higher average wood density and LMA to be associated with broader environmental ranges. To examine the association between species mean traits across sites and elevation, precipitation, and geology ranges, we used an equation identical to Equation (3), with species mean traits rather than ITV as the response variable. The same model structure was retained for this mean trait model as in Equation (3) to allow direct comparison with ITV models.

A second expectation of the alternative hypothesis is that there should be a negative correlation between trait means and across‐site ITV across species if species with more conservative traits are also less plastic. To test this expectation, we calculated Pearson's correlations between site‐level log mean trait values for each species and ITV for both wood density and LMA. Because these analyses use across‐site ITV data, there is only one ITV value for each species.

## 3 Results

**FIGURE 3:**
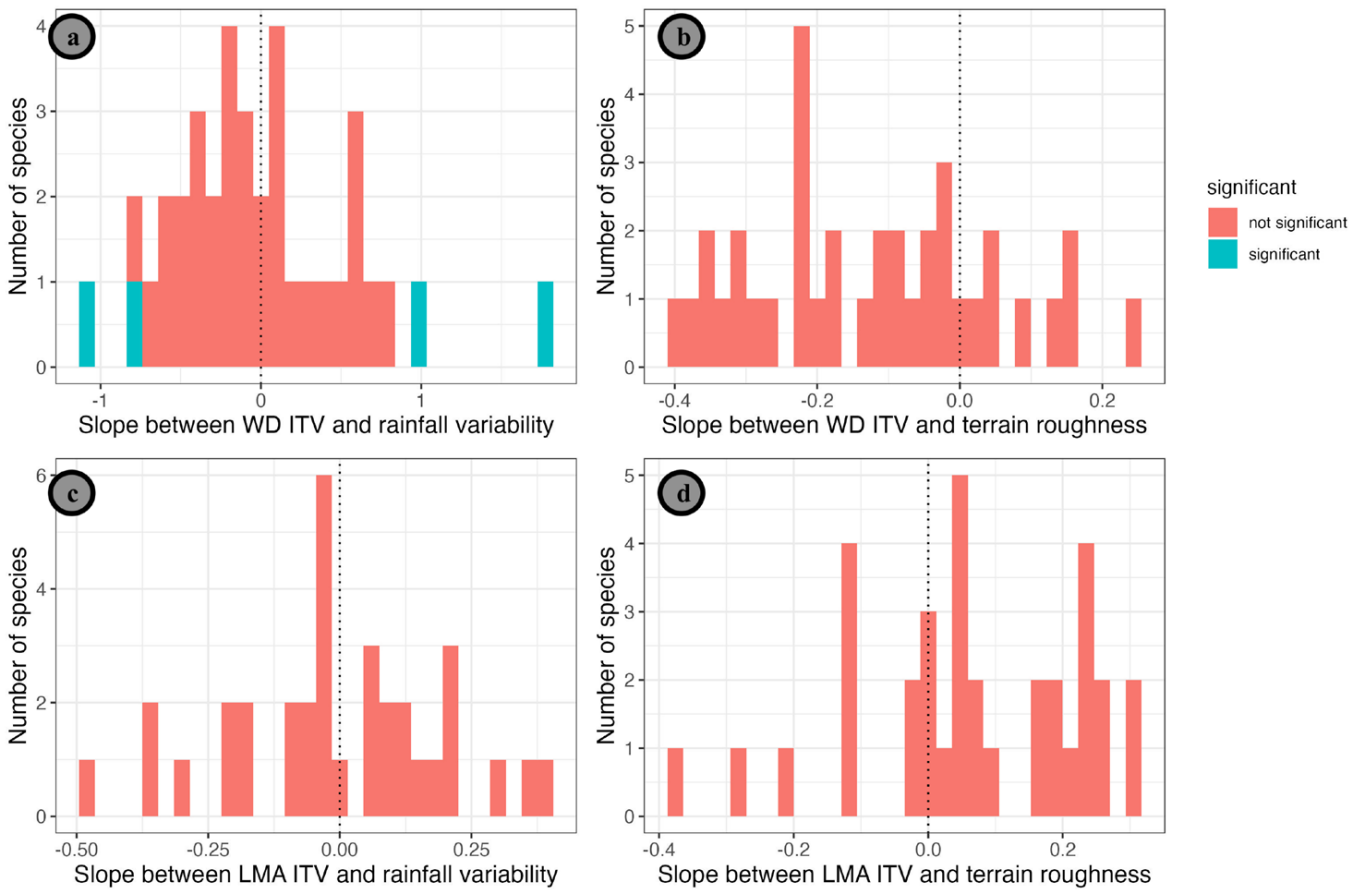
Intraspecific variation in LMA (c & d) and wood density (a & b) against rainfall variability (a & c) and terrain roughness (b & d) for each species ($\beta_{1,\mathrm{sp}}$, Equations 1a and 1b). Species for which the 95% CI does not intersect zero are colored in red; significant slopes are shown in blue. Each bar represents the number of species that had that relationship between ITV* and some terrain or rainfall heterogeneity.

We examined the association between ITV for two key tree traits, LMA and wood density, and site‐ and island‐wide environmental variation. Across all species, we found no significant associations between site‐level terrain roughness (spatial heterogeneity) or rainfall variability (temporal heterogeneity) and within‐site ITV of wood density or LMA ($\beta_{2,\mathrm{sp}}$) (Hypothesis 1), as shown by non‐significant values for $\mu_{2}$ (Table 3, Figure 3). However, rainfall variability was significantly associated with within‐site wood density ITV ($\beta_{2,\mathrm{sp}}$) for four species, but in no consistent direction. Two species, *Bucida buceras* and *Cassipourea guianensis*, showed greater within‐site ITV at sites with high rainfall variability and two, *Coccoloba swartzi* and *Tabebuia heterophylla*, showed lower values (Table A1). None of the species showed a significant association between within‐site ITV for wood density or LMA and terrain roughness.

**TABLE 3:** VIF for Hypothesis 2 (Equation 3).

	MAP range	Elevation range	Irradiance range	PET range	Climatic water index range
WD ITV	1.54	2.59	1.04	2.05	Not used
LMA ITV	Not used	2.360	Not used	2.93	1.65

**FIGURE 4:**
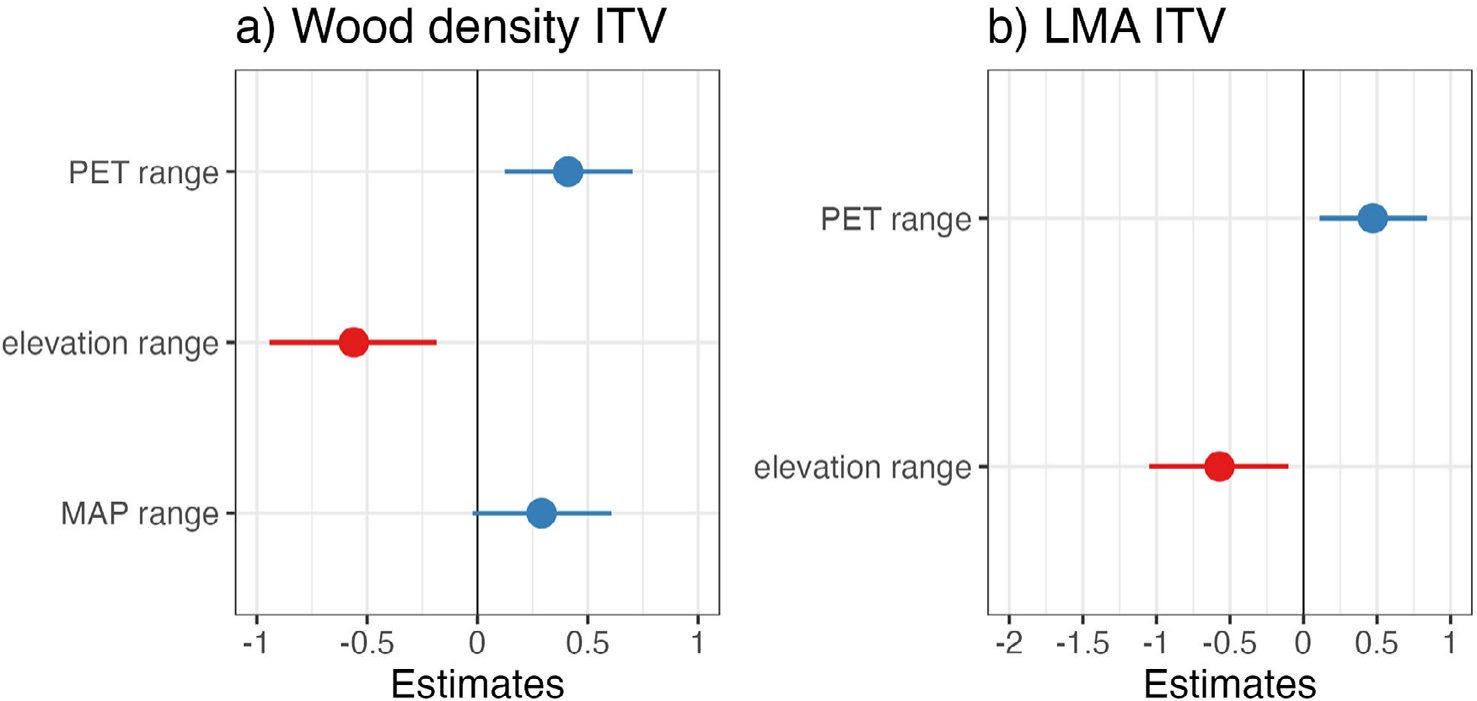
Mean and 95% percentile for credible interval values of slopes for the association between ITV and environmental range across Puerto Rico parameters across all species, (a) ITV* of wood density and (b) LMA. MAP = Mean Annual Precipitation, PET = Potential Evapotranspiration. Negative slopes are plotted in red and positive slopes are plotted in blue. Filled bubbles represent slopes significant at the 95% level.

We also expected a positive relationship between the breadth of environmental conditions in geology, elevation, and mean annual precipitation across species' island‐wide ranges and the magnitude of across‐site ITV (Hypothesis 2), but this was not always the case. Across all species, high across‐site ITV in wood density and LMA were associated with broader PET ranges but a lower range of elevations (Table 4, Figure 4). This relationship is explored in more detail in the discussion. Although the credible intervals for the effect of MAP on across‐site ITV for wood density overlapped 0 (Figure 4a), model comparison showed that including MAP in the model resulted in marginally lower AIC values (Table 3). None of the other environmental variables we considered were significant predictors of across‐site ITV (Table 4).

**TABLE 4:** Estimates of slopes ($\mu_{1}$, Equations (2a), (2b), (2c), (2d)) for the relationships between wood density and LMA ITV and the two metrics of site‐level environmental heterogeneity, rainfall variability and terrain roughness.

ITV*	Terrain variability $\mu_{1}$	Rainfall variability $\mu_{1}$
Wood density ITV*	−0.123 ± 0.137	−0.018 ± 0.153
LMA ITV*	0.057 ± 0.131	−0.075 ± 0.151

**FIGURE 5:**
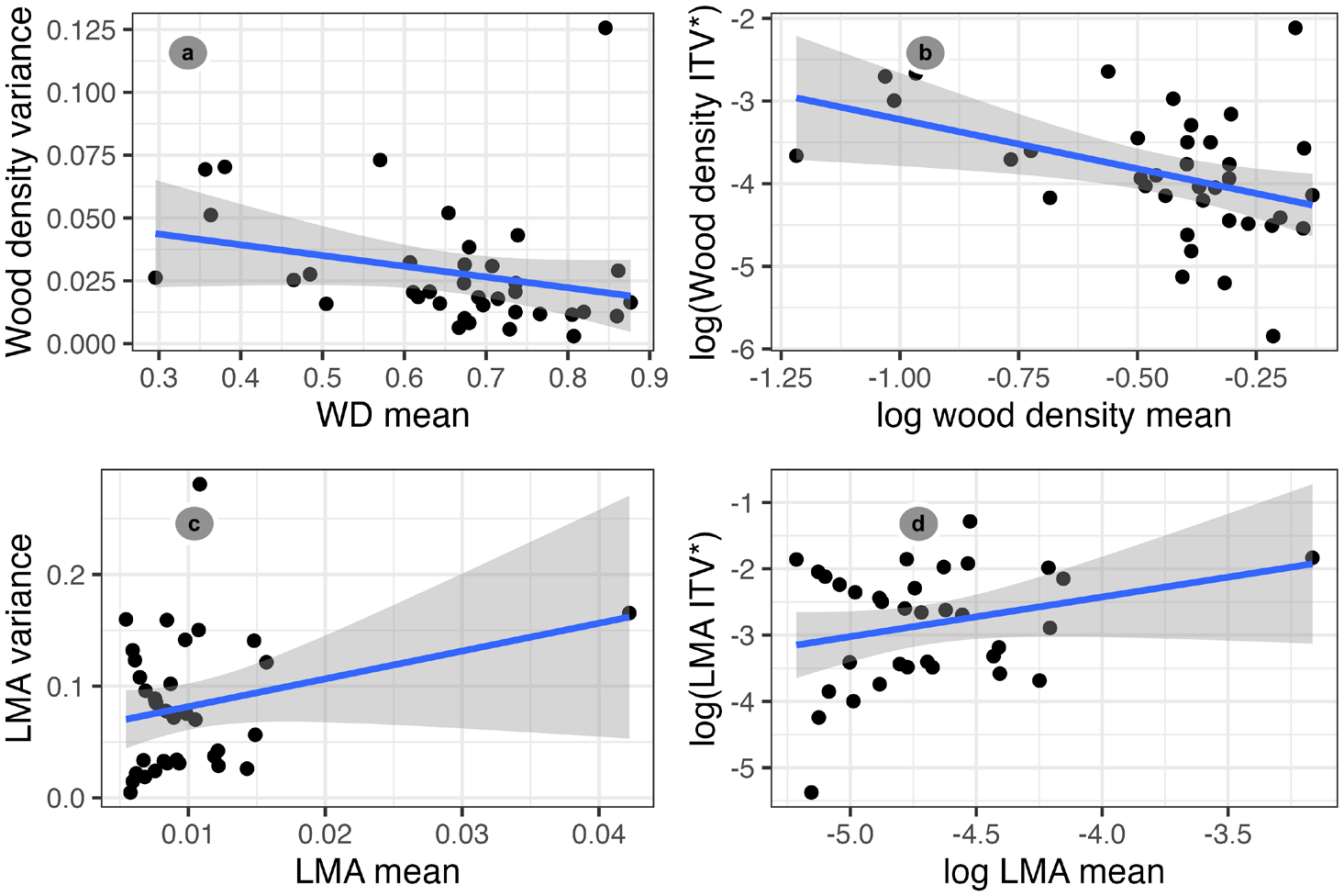
Species‐scale correlations between means and variance for wood density (a, b) and LMA (c, d). Left panels show standard variances. The right panels show logged, Bessel‐corrected variances. Size of each dot corresponds to sample size.

We also tested the alternative hypothesis that species with conservative traits, namely high mean wood density and LMA, might occur across broader environmental ranges (alternative Hypothesis 2) and would exhibit reduced across‐site ITV. Although the relationship was weak, species with high mean wood density had lower across‐site ITV, as expected (Figure 5a–c).

**FIGURE 6:**
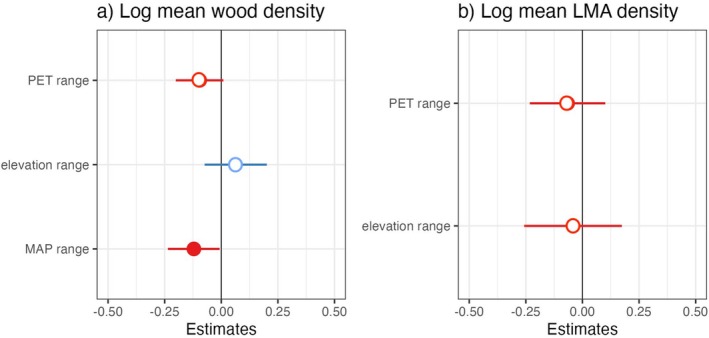
Mean and 95% percentile values of slopes for the association between species mean and environmental range across Puerto Rico across all species. (a) ITV* of wood density shows negative correlations with MAP range, challenging the prediction that conservative species exhibit broader distributions (b) Cloud = cloudiness, CWI = Climatic Water Index, LMA. MAP = Mean Annual Precipitation, PET = Potential Evapotranspiration. Negative slopes are plotted in red and positive slopes are plotted in blue. Filled bubbles represent slopes significant at the 95% level.

However, these species also occurred across a narrower range of mean annual precipitation and PET, contrary to our expectations (Table 4, Figure 6a). The model using mean wood density values also had lower *R* ^2^ than the one for ITV (Table 5). The associations between mean trait values for LMA and island‐wide environmental ranges, however, matched the predictions of the alternative hypothesis for geology, mean annual precipitation, and solar irradiance. Nevertheless, species with high mean LMA occurred across a narrower range of PET and cloudiness (Table 5, Figure 6b). Overall, the model using mean LMA values as a response had a considerably higher explanatory value than the one using ITV (Table 5). Species mean and ITV for LMA were independent (Figure 5d,e), even after accounting for the effect of outliers.

**TABLE 5:** *R* ^2^ and variable selection for models using mean trait and ITV values for wood density and LMA.

	LMA		Wood density
	ITV model		ITV model
*R* ^2^ value for final model	23.75%	*R* ^2^ value for final model	28.18%
AIC for full model	87.19	AIC for full model	85.70
AIC for final model	78.41	AIC for final model	78.25
AIC without PET	82.86	AIC without PET	84.12
AIC without elevation	82.61	AIC without elevation	84.74
		AIC without MAP	79.81

	Mean model		Mean model
*R* ^2^ value for final model	45.01%		23.70%
AIC for full model	23.60	AIC for full model	14.81
AIC for final model	20.13	AIC for final model	7.60
AIC without PET	23.56	AIC without PET	10.60
AIC without geology	22.35	AIC without MAP	11.26
AIC without MAP	24.46		
AIC without irradiance	28.80		
AIC without cloud cover	26.05		

## 4 Discussion

Our central question was how ITV is structured along different scales of environmental variation. We examined this question at the site and range scales and used ITV in two traits, LMA and wood density, for 33 tree species in eight sites across Puerto Rico. Contrary to our expectations, we found no significant relationship between within‐site ITV and within‐site environmental variation. However, high across‐site ITV was associated with broader ranges across some environmental variables. We found mixed support for our alternative hypothesis that higher values of LMA and wood density (reflecting more conservative resource strategies for light and water, respectively) (Westoby et al. 2004; Díaz et al. 2016; Kunstler et al. 2016) would be associated with larger environmental ranges.

Contrary to our first hypothesis, that within‐site ITV would be higher in environments with more within‐site topographic or rainfall seasonality, we found no significant associations with either wood density or LMA ITV across all species. Though plants can exhibit plasticity in response to environmental heterogeneity (Bin et al. 2022; Baythavong 2011; Anderegg et al. 2021), most existing studies of ITV in trees have compared trait expression across regional environmental gradients (e.g., Anderegg et al. 2021; Fajardo et al. 2024) rather than within sites. At the site level, some studies have reported a lack of association between ITV and site‐level variables (Kang et al. 2014; but see Anderegg et al. 2021). Another study of tropical trees also found that within‐site ITV for wood density was poorly explained by micro‐environmental heterogeneity (e.g., topography) (Zuleta et al. 2022), although the same study found environmentally structured variation for specific leaf area (the inverse of LMA) for some species. Although these sites exhibited substantial variation in terrain variability and rainfall seasonality (see Table 1), these environmental variables may not correspond to the scale at which traits vary for these tropical tree species. The simultaneous action of multiple forces that generate ITV makes it inherently difficult to predict patterns at the within‐site level.

We also examined the relationship between across‐site ITV and environmental range size. Trait variability has been hypothesized to limit species' realized niche (Laforest‐Lapointe et al. 2014), leading to the common empirical result of higher ITV in species that occupy larger geographic ranges (Rixen et al. 2022). Indeed, higher across‐site wood‐density ITV was significantly associated with broader ranges of PET and (marginally) MAP but lower ranges of elevation. Water availability is a strong environmental filter that selects against species with less dense wood (Mo et al. 2024). Our observation that species with high ITV wood density occur across wide ranges of water availability (PET and MAP) therefore aligns with our hypotheses. Although the negative association with elevation range may seem counterintuitive, it likely emerges because of the specific range of climatic conditions (PET and MAP) experienced by species that typically occupy low and high elevation areas. In particular, elevational changes in low coastal areas in Puerto Rico (especially on well‐drained limestone soils) may be associated with more biologically significant shifts in climatic conditions compared to equivalent elevational ranges in higher montane areas. A study comparing functional tree diversity across volcanic and limestone forests in Puerto Rico (Muscarella et al. 2020) found a negative association between fine‐scale topographic heterogeneity and wood density diversity across species in the driest, limestone sites, suggesting that environmental stress at these sites may constrain trait expression within species. Another study on these same plots found the lowest levels of community‐level functional diversity of wood density in dry limestone plots (Muscarella et al. 2016). Overall, these results highlight the idea that understanding spatial variation of biodiversity (ITV in the current study) requires assessing the environmental conditions experienced by organisms in geographic space, including the physiological relevance of the conditions (Graham et al. 2025).

We found mixed support for the alternative hypothesis that species with more conservative resource strategies (i.e., those with high wood density and LMA) might have broader environmental ranges. We hypothesized that conservative species are adapted to harsh environments and may be less plastic in response to environmental variation, leading to lower ITV (Valladares et al. 2007; Markesteijn and Poorter 2009; Anderegg et al. 2021). In contrast to our expectations, species with high wood density occurred across a narrower absolute range of precipitation and PET, suggesting that species with hard wood are restricted to relatively dry sites (Muscarella and Uriarte 2016). This finding contrasts with some predictions of the conservative–acquisitive strategy framework (Reich 2014) and suggests that highly conservative species may instead be confined to more stable, stress‐prone habitats with limited climatic fluctuation. In contrast, species with higher values of LMA existed across both geologies and a broader range of precipitation and solar irradiance, perhaps indicating weaker trait filtering for this trait compared to wood density. As above, however, it is important to consider the biological significance of the range of conditions experienced by species that tend to occupy different areas.

Many authors have emphasized the importance of incorporating trait variation in ecology for determining species distributions across their environmental ranges (Holyoak and Wetzel 2020; Siefert 2012), but few offer suggestions on how ITV should be structured across these ranges. To properly understand ITV and its consequences for species responses to environmental change, we must account for the multiple forces at play that constrain variation. On the one hand, we found broad support for the notion that species with greater environmental breadth express higher levels of ITV. However, the environmental breadth of occurrence was also narrower for species with high wood density, a conservative trait value. Our findings underscore that ITV is structured more strongly by broad‐scale environmental gradients than by local heterogeneity, challenging a common assumption in trait‐based ecology. Our results also reveal how ITV can be constrained in conservative species while acquisitive species would be more able to shift range using their higher ITV, highlighting the need for a more nuanced understanding of the ways in which species may respond to varying environmental conditions.

## Author Contributions

**Eva Arroyo:** conceptualization (equal), formal analysis (equal), methodology (equal), project administration (equal), software (lead), visualization (lead), writing – original draft (lead), writing – review and editing (equal). **Robert Muscarella:** data curation (equal), supervision (supporting), writing – review and editing (supporting). **María Uriarte:** conceptualization (lead), data curation (equal), supervision (lead), writing – original draft (supporting), writing – review and editing (supporting).

## Conflicts of Interest

The authors declare no conflicts of interest.

## Supporting information


**Data S1:** ece372224‐sup‐0001‐supinfo.docx.

## Data Availability

All code and data are accessible via Zenodo: https://zenodo.org/records/16915359.

## Appendix A

**TABLE A1:** ITV values and environmental range for each species used, as well as Pearson's correlations with significance levels for environmental gradients.

Species name	ITV wood density	ITV LMA	Mean annual precipitation range	Geology occurrence (*L* = limestone, *V* = volcanic)	Elevation range	Geology correlation		MAP correlation		Elevation correlation	
						Mean WD	Mean LMA	Mean WD	Mean LMA	Mean WD	Mean LMA
*Alchornea latifolia*	0.070	0.096	2627.83	Both	671.10	−0.87*	−0.72	0.13	−0.71	−0.49	0.16
*Andira inermis*	0.031	0.132	1713.26	Both	570.73	−0.23	−0.86	−0.15	−0.62	−0.09	0.36
*Ardisia obovata*	0.012	0.121	655.85	Both	545.60	NA	NA	0.65	0.81	0.83	0.94
*Bucida buceras*	0.126	0.076	1738.97	L	820.70	NA	NA	−0.8	−1*	−0.94	−0.64
*Calophyllum antillanum*	0.021	0.281	1249.97	Both	310.24	−0.76	−0.82	−0.9*	−0.3	−0.19	0.86
*Casearia arborea*	0.032	0.108	1119.01	Both	394.10	NA	NA	−0.08	−0.76	0.14	0.66
*Casearia sylvestris*	0.010	0.019	1911.12	Both	538.68	−0.34	0.59	−0.22	0.28	−0.93**	0.49
*Cecropia schreberiana*	0.026	0.159	2109.72	Both	764.00	−0.9**	−0.95**	−0.78	−0.67	−0.15	−0.73
*Clusia rosea*	0.021	0.166	1934.07	Both	696.80	NA	NA	NA	NA	NA	NA
*Coccoloba pyrifolia*	0.031	0.029	2761.90	Both	804.26	−0.16	0.99	−0.76	0.86	−0.84	−0.24
*Coccoloba swartzii*	0.038	0.141	2799.11	Both	763.12	−0.79	0.02	−0.3	0.72	−0.11	−0.32
*Coffea arabica*	0.006	0.005	NA	NA	NA	NA	NA	0.1	−0.69	0.47	0.52
*Cassipourea guianensis*	0.012	0.022	2117.13	V	821.86	NA	NA	−0.99*	−0.44	0.86	0.08
*Daphnopsis philippiana*	0.069	0.033	1320.05	V	386.39	NA	NA	−0.83	0.99*	0.98	−0.87
*Dendropanax arboreus*	0.025	0.160	555.61	Both	708.75	−0.74	−0.3	−0.44	−0.18	−0.89	0.7
*Drypetes glauca*	0.008	0.024	2159.85	V	579.60	NA	NA	0.88	−0.05	−0.99*	0.42
*Eugenia monticola*	0.011	0.150	NA	Both	NA	−0.5	−0.47	−0.63	−0.2	−0.7	−0.53
*Eugenia stahlii*	0.006	0.042	1946.46	Both	739.00	NA	NA	0.02	−0.58	0.83	0.87
*Faramea occidentalis*	0.024	0.123	2420.85	Both	662.00	−1**	−0.96	−0.98	−0.88	0.31	0.57
*Guapira fragrans*	0.020	0.078	1205.69	L	845.20	NA	NA	−0.2	0.86	−0.46	0.68
*Guarea glabra*	0.073	0.034	2609.01	V	568.85	NA	NA	−0.31	−0.81	0.96**	0.66
*Inga laurina*	0.016	0.102	2164.98	V	547.60	NA	NA	0.37	−0.7	−0.14	0.31
*Matayba domingensis*	0.013	0.026	1272.38	V	462.25	NA	NA	0.96	0.03	−0.79	0.34
*Micropholis guyanensis*	0.018	0.056	2422.31	V	834.20	NA	NA	−0.7	−0.49	−0.31	0.6
*Myrcia deflexa*	0.003	0.034	1042.06	Both	261.70	NA	NA	0.74	−0.58	0.39	0.79
*Neolaugeria resinosa*	0.015	0.072	2172.93	Both	832.80	NA	NA	−0.33	0.71	−0.58	0.48
*Ocotea leucoxylon*	0.028	0.031	2162.95	Both	490.54	−0.09	−0.61	−0.72	−0.34	−0.06	−0.2
*Pseudolmedia spuria*	0.018	0.015	1718.33	Both	389.00	−1**	−0.41	−0.86	−0.84	0.87	−0.06
*Sideroxylon salicifolium*	0.013	0.070	2157.61	L	739.90	NA	NA	0.37	−0.44	0.97	0.49
*Sloanea berteriana*	0.043	0.031	553.71	V	266.42	NA	NA	−0.22	−0.83	0.95*	0.45
*Syzygium jambos*	0.019	0.037	2591.79	V	781.63	NA	NA	−0.52	−0.31	−0.04	−0.03
*Tabebuia heterophylla*	0.052	0.141	NA	Both	NA	−0.95*	−0.29	−0.73	−0.66	0.56	0.33
*Thouinia striata*	0.029	0.085	1019.74	L	278.50	NA	NA	0.91	0.56	0.76	0.31

* *p* < 0.10.

** *p* < 0.05.
