# Correction to “Intraspecific Trait Variation in Tree Species Responds to Environmental Heterogeneity at Range‐Wide but Not Local Scales”

**DOI:** 10.1002/ece3.72524

**Published:** 2025-11-14

Arroyo, E., R. Muscarella, and M. Uriarte. 2025. “Intraspecific Trait Variation in Tree Species Responds to Environmental Heterogeneity at Range‐Wide but Not Local Scales.” *Ecology and Evolution* 15, no. 10: e72224.

The MAP and Geology columns in Table 1 of the published article contained several errors. The corrected table is provided below.

**Table:** 

Site	MAP	Geology	Elevation (m)	Rainfall variability	Topographic variability	# Species wood density	# Species for LMA
Cambalache (CAM)	1545.40	Limestone	82.00	28.31	5.40	12	10
Carite (CAR)	1861.90	Volcanic	577.90	38.25	2.85	15	15
Guajataca (GUA)	1728.50	Limestone	221.50	41.71	6.24	14	12
Guanica (GUN)	1039.30	Limestone	878.30	54.12	5.61	20	19
Guilarte (GUI)	2220.06	Volcanic	186.00	59.41	3.13	5	3
Luquillo (LUQ)	2351.09	Volcanic	1090.80	26.85	3.59	28	27
Rio Abajo (RIO)	2068.96	Limestone	317.00	41.61	7.12	15	15
Toro Negro (TOR)	2139.76	Volcanic	887.30	44.07	3.62	19	19

This error does not affect the underlying data, analyses, or conclusions of the paper.

We apologize for this error.

