# Supplementary material for: Intraspecific Trait Variation in Tree Species Responds to Environmental Heterogeneity at Range‐Wide but Not Local Scales
Source: Ecol Evol. 2025 Sep 28;15(10):e72224. doi: 10.1002/ece3.72224 (PMC12476926; doi:10.1002/ece3.72224)
Supplement: Supplementary file 1 — Data S1: ece372224‐sup‐0001‐supinfo.docx. [file ECE3-15-e72224-s001.docx]

***Supplemental figures and information:***

We used the variance inflation factor (VIF) to analyze the models fit in Eqn. 7 around how ITV relates to environmental range. VIF over 5 indicates multi-collinearity, that the way the predictors relate to the response variables should confound results. No variables used showed multicollinearity with respect to the VIF (SI Table 1). Additionally, we calculated Pearson’s correlations between the range predictors (SI Figure 1). Most are lower than 0.6, with the exception of the correlation between Elevation range and PET range (*r*=0.75). Although we recognize that it may be difficult to separate the effect of these two variables in practice, we included both in the same model based on low VIF.

**TABLE S1:** VIF for Hypothesis 2, Equation 7.

|  | MAP range | Elevation range | Irradiance range | PET range | Climatic water index range |
| --- | --- | --- | --- | --- | --- |
| WD ITV | 1.544 | 2.590 | 1.038 | 2.048 | Not used |
| LMA ITV | Not used | 2.360 | Not used | 2.929 | 1.648 |


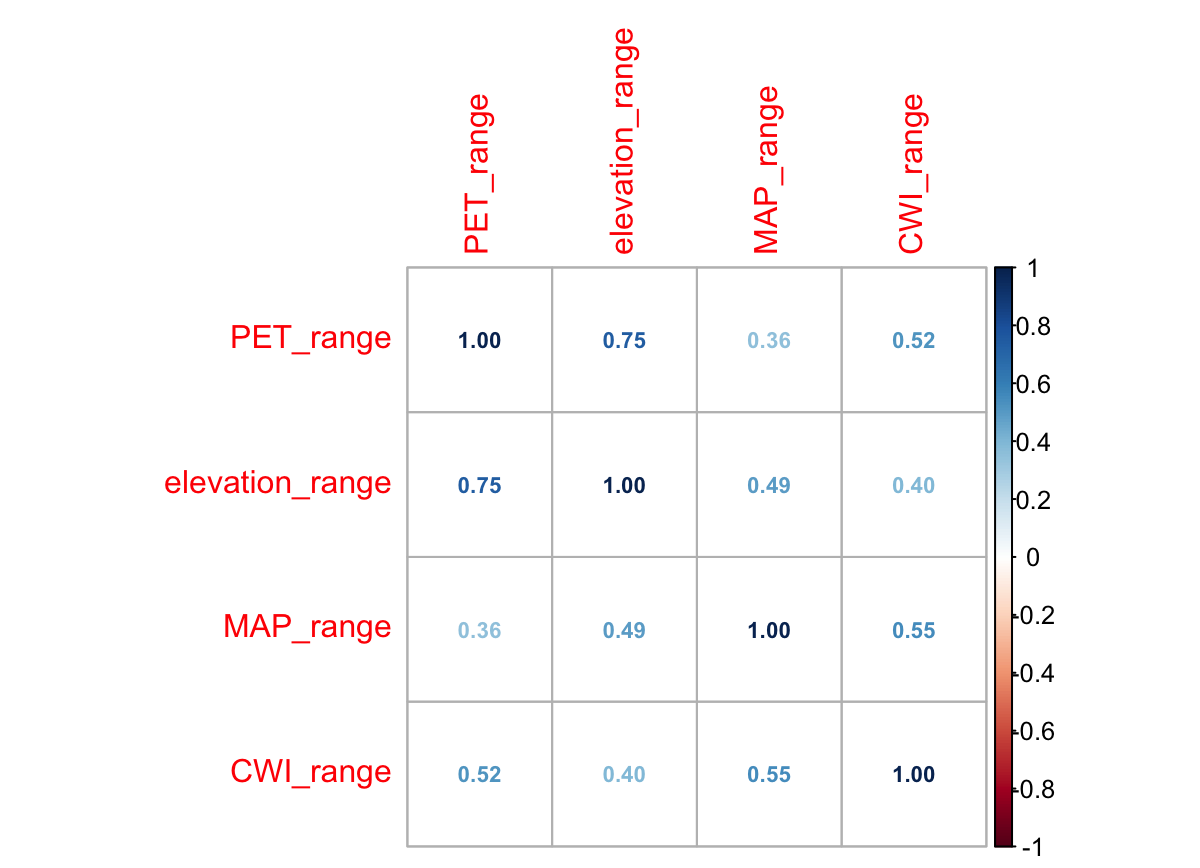


SI Figure 1: Correlations between the environmental range variables.
